# A Study on the Influence of Authoritarian-Benevolent Leadership on Employees' Innovative Behavior From the Perspective of Psychological Perception—Based on Fuzzy Set Qualitative Comparative Analysis

**DOI:** 10.3389/fpsyg.2022.886286

**Published:** 2022-06-02

**Authors:** Lingxi Meng, Tong Li, Mengyuan Yang, Shanshan Wang

**Affiliations:** ^1^School of Management, Yunnan Minzu University, Kunming, China; ^2^School of Finance, Yunnan University of Finance and Economics, Kunming, China; ^3^Yunnan College of Tourism Vocation, Kunming, China

**Keywords:** authoritarian-benevolent leadership, employees' innovative behavior, psychological perception, qualitative comparative analysis, China's situation

## Abstract

Employee innovation is the key to enhancing the core competitiveness of an enterprise, and leadership style plays an important role in stimulating employees' innovative behavior. This study explores the impact of unique ambidextrous leadership in the Chinese context, authoritarian-benevolent leadership, on employees' innovative behavior from the perspective of employees' psychological perception, based on research data from 430 employees of companies with direct leaders. Based on the configuration theory, using the fuzzy set qualitative comparative analysis method, the configuration analysis was carried out by taking authoritarian-benevolent ambidextrous leadership and employees' psychological perception as the influencing factors and obtained five configurations of high-level employees' innovative behavior. The results show that the combination of individualized care, understanding, and forgiving of benevolent leadership and Shang-yan of authoritarian leadership can effectively stimulate employees' innovative behavior. Juan-Chiuan leadership is not conducive to employees' innovative behavior. Employees' high psychological safety and low uncertainty are important conditions for promoting employee innovation. In this study, the four dimensions of authoritarian-benevolent leadership and the psychological perceptions of employees are discussed in combination, and the paths of motivating employees to innovate actively are obtained. It is hoped that it can provide certain ideas for leaders to promote employee innovation.

## Introduction

Innovation is an eternal topic. In the early twentieth century, Joseph Alois Schumpeter proposed that innovation is the most important driver of economic development. Employees' innovative behavior refers to the process by which employees generate, introduce, and apply beneficial novel ideas or things in organization-related activities, including forming or developing new ideas or technologies, changing existing management procedures to improve work efficiency, etc. (Liu and Shi, 2009). Enterprise innovation is an organic combination of employees' innovative behavior and the development of an enterprise depending on the support of employees' innovative behavior. Employee innovation is an important source of enterprise innovation and the key to maintaining vitality and advantages in complex environments and increasingly fierce competition. How to better stimulate employees' innovative behavior has become the focus of academic and practical circles.

The influence of leadership style on employees' innovative behavior is a classic and important research topic in academia and practice (Lu et al., 2021). Leadership style is an important factor affecting employees' innovative behavior (Zhang and Bartol, 2010). Previous studies have focused on the impact of a single type of leadership style on employees' innovative behavior. However, the internal and external environment of today's organizations is dynamic and changeable, and it is difficult for a single leadership style to meet the diverse needs of the organization and its members (Zhang et al., 2015). Therefore, in recent years, ambidextrous leadership has gradually attracted the attention of scholars. Ambidextrous leadership refers to the behavior of leaders using “both-and” thinking, combining specific situations to switch between two opposing and complementary leadership styles, and flexibly deal with organizational problems (Rosing et al., 2011). Studies on ambidextrous leadership include cognitive perspective (Rosing et al., 2011), entitlement perspective (Sagie et al., 2002), routine perspective (Bass et al., 2003; Schreuders and Legesse, 2012), spatio-temporal perspective (Li et al., 2020), and authoritarian-benevolent perspective (Nan and Jian, 2019). Looking back at the literature, most of the past ambidextrous leadership research is in the context of western culture. If these theories are used to discuss employee innovation behavior in the Chinese context, there may be cross-cultural problems. However, authoritarian-benevolent leadership of ambidextrous leadership is widespread in China, but there is still a lack of research on the impact of this ambidextrous leadership on employees' innovative behavior, which needs to be explored urgently. Therefore, this research will select authoritarian-benevolent leadership of ambidextrous leadership, which is shaped by Chinese Confucian traditional culture.

Authoritarian-benevolent leadership is a unique ambidextrous leadership in the Chinese context. Under the influence of traditional Chinese culture, the relationship between the superior and the subordinate of the organization will be like that of “monarch and minister” and “father and son.” Leaders will show both a benevolent side and an authoritarian side, the purpose of which is to achieve the management effect of making subordinates grateful, reverent, and obedient. At present, several studies have found the positive value of authoritarian-benevolent leadership. Nan and Jian (2019) found through empirical research that when the leader's authoritarian-benevolent leadership is balanced and the levels are high, it can stimulate the active execution of subordinates, thereby improving the work performance. Liu and Guo (2021) found that authoritarian-benevolent leadership has a significant negative impact on employees' job withdrawal behavior. However, there is also some research suggesting that this leadership style may not always lead to positive effects. A high level of authoritarian-benevolent leadership will positively affect the uncertainty of subordinates, thereby reducing their subjective wellbeing (Nan and Jian, 2019). Authoritarian-benevolent leadership will also have a potential negative impact on employee trust (Jiao, 2022). From the above contradictions, some urgent questions need to be explored: Is authoritarian-benevolent leadership a positive leadership style? Can it positively influence employees' innovative behavior? This study argues that since benevolent leadership includes two dimensions of individualized care and understanding and forgiving (Farh and Cheng, 2000), and authoritarian leadership includes two dimensions of Juan-Chiuan and Shang-yan (Chou et al., 2010), so although they all adopt authoritarian-benevolent leadership, different leaders may have different combinations of dimensions, resulting in different effects on the innovative behavior of subordinates.

In addition, the formation process of employees' innovative behavior is very complicated, so the research should not only consider the unilateral influence of the leader, but also the subordinate's factors, especially the psychological perceptions. People's psychology determines their behaviors, and their behaviors are the embodiments of their psychology. As psychological states, employees' psychological safety and uncertainty are highly related to their innovative behaviors as well as their leaders' styles. Specifically, Carmeli et al. (2009) found that psychological safety has a positive impact on individual behaviors such as learning, innovation, and advising. In an organizational environment with high difficulty and high risk, the psychological safety of employees is regarded as a prerequisite for innovative behavior (Nembhard and Edmondson, 2006). Psychological safety can bring good psychological experience to employees, which can improve employees' learning ability, and make them willing to try new ideas to explore and participate in creative work to improve creativity. Benevolent leaders bring psychological safety to employees through personal care for their subordinates as well as support and resources at work (Su and Liu, 2018). Authoritarian leadership emphasizes the leader's absolute control over employees and absolute authority over self, which will increase employees' psychological pressure, and high power distance will reduce their psychological safety (Peng and Shuangli, 2018). The uncertainty of employees will jeopardize their self-evaluation (Lian et al., 2012) and their sense of control over the work environment, making them doubt their ability to complete a certain task, which is not conducive to the generation of employees' innovative behavior. In addition, De Cremer (2003) argues that subordinates experience more uncertainty when leaders exhibit inconsistent behaviors before and after. Zhao and Guo (2017) also pointed out that the two behaviors of leaders showing differences may lead to a cognitive dissonance of subordinates, thus deepening their uncertainty.

To sum up, this study will take the four dimensions of authoritarian-benevolent leadership, the psychological safety and uncertainty of employees as the influencing factors, and explore its influence on employees' innovative behavior. At this time, employees' innovative behavior is a complex result of the joint action of many factors. However, trapped in the mindset of linear causality, most of the existing related research is the analysis of the influence mechanism or mediating effect of a single factor on employees' innovative behavior. These studies analyzed the net effect of authoritarian-benevolent leadership as a single variable and analyzed the psychological perception of employees as an intermediary variable. It is a holistic and diverse perspective that needs to be adopted. Therefore, based on the configuration theory, this study will use the qualitative comparative analysis method (Ragin, 1987) to carry out research to find out the configurations that can stimulate employees' innovative behavior. This has important theoretical significance, and can also provide a certain reference for leaders to manage employee innovation.

## Theory and Model

### Authoritarian-Benevolent Leadership

Authoritarian-benevolent leadership is a leadership style with local Chinese characteristics. Nan and Jian (2019), based on the perspective of ambidextrous leadership, define authoritarian-benevolent leadership as leaders who possess two complementary leadership behaviors of benevolence and authoritarianism and can coordinate and use these two leadership behaviors according to the situation. Leaders who adopt this style are not only sympathetic to their subordinates and concerned about their work needs (Farh and Cheng, 2000) but also require subordinates to be highly obedient to achieve high work performance (Chou et al., 2010). Today, authoritarian-benevolent leadership is still widespread in Chinese enterprise management.

### Benevolent Leadership

Benevolent leadership refers to the leader's long-term concern for subordinates' work and personal wellbeing (Cheng et al., 2000). Benevolent leadership includes two aspects: individualized care and understanding and forgiving: Individualized care is mainly reflected in life, which means that leaders endow subordinates with favors to create a comfortable and supportive work environment; understanding and forgiving are mainly manifested at work, which means that even if a subordinate occasionally makes a mistake or mishandles a problem, the leader can understand the reason behind it and help when the subordinate needs help. At present, studies have shown that benevolent leadership can reduce subordinates' work pressure and work alienation by providing more care and resource support (Lirang et al., 2014), improve subordinates' work attitude and work performance (Peng et al., 2016), improve the loyalty and work effort of subordinates (Shin et al., 2012), and promote the creativity of employees (Wang and Cheng, 2010). However, some studies have pointed out that leaders blindly showing benevolent leadership can easily cause subordinates to breed inertia, and even violate the rules (Li et al., 2015). Xia (2020) also found that benevolent leadership limits employees' innovative performance through subordinates' order obedience.

### Authoritarian Leadership

Farh and Cheng (2000) define authoritarian leadership as a leadership behavior in which a leader emphasizes his or her absolute authority, tightly controls subordinates, and demands unreserved obedience from them. Previous studies have shown that authoritarian leadership is a typical leadership style characterized by control, while a controlling leadership style is mostly regarded as a negative leadership style that will weaken subordinates' work motivation. However, many scholars think about the reason and meaning of authoritarian leadership and think that only using “control” to summarize authoritarian leadership cannot clarify its complete connotation. Chou et al. (2006) found that authoritarian leadership includes two aspects: Juan-Chiuan and Shang-yan. To maintain their dignity, Juan-Chiuan leaders often depreciate their subordinates' abilities, deliberately criticize and ignore their contributions, and make their subordinates obey through manipulation. Their style can easily reduce subordinates' job satisfaction (Cheng et al., 2004), weaken their positive emotions or attitudes, and even lead to negative behaviors (Qi et al., 2020). Shang-yan leaders will monitor subordinates' work tasks according to standards and principles, and require subordinates to achieve goals and abide by organizational norms, to promote subordinates to produce high work results. Chou et al. (2010) pointed out that the focus of control is different between Juan-Chiuan leadership and Shang-yan leadership. The control objects of Juan-Chiuan leadership are people, and the control objects of Shang-yan leadership are things.

### Employees' Psychological Perception

#### Psychological Safety

Psychological safety refers to an individual perception that it will be safe to express personal opinions, suggestions, and concerns in an organization without being punished, criticized, or treated unfairly (Liang et al., 2012). Kahn (1990) believes that when employees have a high level of psychological safety, they will perceive that the external environment is safe, realize that they can express their true thoughts without being excluded and hurt, and dare to show themselves boldly, to show higher initiative. He also pointed out that a leader with a positive style can improve the psychological safety of employees. Similarly, Edmondson (1999) believes that a supportive leadership style can effectively improve employees' psychological safety. Liang et al. (2019) also pointed out that in the context of Chinese organizations, the behavior of subordinates is often closely related to the conduct and style of leaders. A good relationship between superiors and subordinates can give employees a high level of psychological safety and encourage employees to let go to do what they think is good for the organization.

#### Uncertainty

Uncertainty is people's general perception of work uncertainty which reflects the general perception that an employee lacks the information or situational understanding needed to accurately predict the future (Colquitt et al., 2012). Uncertainty is related to uncertainty management theory. Hou et al. (2019) introduced uncertainty management theory to explore how to mitigate the uncertainty caused by authoritarian-benevolent leadership to subordinates. Colquitt et al. (2012) created a measure to assess employees' uncertainty based on this theory, which includes the inability to predict or control future events, the existence of environmental volatility, and a sense of environmental complexity or heterogeneity. And the items in the research were not referenced to any particular target, because the theory clearly states that its focal construct is very broad and general. Uncertainty will bring potential psychological pressure to employees, and in the process of facing and dealing with pressure, subordinates often consume a lot of psychological resources (Chiu et al., 2015), resulting in their low level of job satisfaction and low motivation emotional experience. Colquitt et al. (2012) found that uncertainty is negatively correlated with job performance and had an important predictive effect on job performance.

#### Employees' Innovative Behavior

Innovation is the soul of a nation's progress, and it has become the research object of many scholars (Chin et al., 2021; Duan et al., 2021, 2022), who study the internal mechanism of innovation from the perspectives of knowledge hiding, strategic risks, and Asia-Pacific business models. Liu and Shi (2009) defined employees' innovative behavior as the process of generating, introducing, and applying beneficial novel ideas or things in organizational activities, including forming or developing new ideas or technologies, changing the existing management procedures to improve work efficiency, etc. This definition is based on research by Scott and Bruce (1994). Scott and Bruce (1994) believed that employees' innovative behavior starts from the individual's cognition of the problem and the formation of ideas, and goes through multiple stages in which innovative individuals seek assistance with their ideas, try to build a supporter alliance, put innovative ideas into practice, build innovative prototypes or models, and finally form commercialized products or services. It is a complex process involving the generation, promotion, and practice of ideas. The characteristic of the innovation process is that it is a combination of a series of discontinuous activities rather than a discrete sequential process. It has multiple stages, with different activities and innovative behaviors in different stages, and individuals can participate in these behaviors at any time. Employees' innovative behavior is neither the expected role behavior of employees nor a clear corporate vision formed by employees and the organization. Innovative behavior is completely an out-of-role behavior that is freely determined by employees and is not recognized by the organization's reward system. However, employees' innovative behavior is conducive to the organization, the group, and even their effective completion of tasks (Janssen, 2000). Relevant studies have shown that the leadership style of superiors (Dess and Picken, 2000) and the psychological perceptions of employees are important factors that affect employees' initiative innovation.

### Authoritarian-Benevolent Leadership, Employees' Psychological Perception, and Employees' Innovative Behavior

As a complex leadership style, authoritarian-benevolent leadership includes two kinds of leadership styles: benevolent and authoritarian, each of which includes two dimensions, respectively. The influence mechanism of this style of leadership is complex. A complex mechanism exists between authoritarian-benevolent leadership, moral disengagement, and unethical pro-organizational behavior (Shaw et al., 2020). Benevolent leaders will give subordinates a “soil” for innovation by creating a suitable working atmosphere and providing necessary help, which is conducive to promoting employees' active innovation. But the sense of loyalty and obedience inspired by benevolent leadership also affects subordinates. Driven by this role responsibility, employees may not propose alternative problem-solving strategies, but just blindly accept orders from leaders, hinder their unique thinking, and thus inhibit their creative play (Zhang et al., 2013). On the other hand, authoritarian leaders will establish authority and strictly control subordinates, which may reduce the possibility of employees exhibiting out-of-role behaviors and inhibit the emergence of employee innovative behaviors (Chen et al., 2014). However, it may also lead to high self-requirements and work engagement of subordinates, further enhancing employees' sense of organizational identity (Xiangying and Guibin, 2018), thereby improving their subjective initiative, and promoting employees' innovative behaviors to a certain extent. Xu et al. (2022) studied authoritarian leaders in high-tech enterprise teams and found that the effect of authoritarian leadership on creative deviance is complex. When the level of authoritarian leadership is low, it will promote creative deviance, and when the level of authoritarian leadership is high, it will inhibit creative deviance behavior. Zhang et al. (2021) found that authoritarian leadership can positively influence employee innovation behavior within a certain range. To sum up, authoritarian-benevolent leadership must be highly related to employees' innovative behavior, but at present, there is little research on the influence of this kind of ambidextrous leadership, which is widely present in Chinese organizational management, on the innovative behavior of employees. This study argues that it is precisely because benevolent leadership and authoritarian leadership of this leadership can be divided into two dimensions, forming a combination of different dimensions, so it has a differentiated effect on employees' innovative behavior. Based on the perspective of configuration, it is necessary to subdivide the leadership into four dimensions and explore its influence on the innovative behavior of employees.

In the formation process of employees' innovative behavior, employees' psychological perceptions also play a great role. The empirical research of Wang et al. (2021) found that leaders' psychological capital has a significant positive impact on employees' innovative behavior, and psychological safety has a partial mediating effect between the two. Wang et al. (2018) conducted an empirical study on 106 teams and found that psychological safety has a mediating effect between humble leadership and employees' innovative behavior. Unlike other behaviors in the workplace, innovation is a process of exploring the unknown from the known and may face the danger of failure at any time (Jiang et al., 2019). In addition, innovation is also a subversion of existing technologies, processes, and norms to a certain extent, which may attract resistance and opposition. Therefore, when employees have a high level of psychological safety, the psychological pressure brought by innovation risks will be weakened, which will make employees not afraid of the possible risk consequences of innovation failures, and dare to break through the existing thinking and work frameworks, to be more engaged in innovation (Chen et al., 2014; Liu et al., 2019). When employees have a high level of uncertainty, employees will feel confused and worried about the work situation, doubt their abilities, and even reduce their trust in the organization and leaders, which will accelerate the exhaustion of resources. In addition, a high level of uncertainty will also make employees feel the pressure from the work itself, that is, concerns about job stability, resulting in a sense of insecurity (Huang et al., 2010), causing their concerns about personal needs and achievement of goals (Baumeister and Leary, 1995; Eberly et al., 2017), unease and apprehension about the *status quo*. These factors are not conducive to employees' innovation when faced with problems at work. It can be seen that the psychological safety and uncertainty of employees are important antecedent variables of their innovative behavior.

In addition, the psychological perceptions of subordinates are not only related to their characteristics and work situation factors but also are often closely related to the style and behavior of leaders. Authoritarian-benevolent leadership has an impact on employees' psychological safety and uncertainty. Specifically, benevolent leaders will show comprehensive and long-term care and support for the wellbeing of employees, which can make employees feel recognized and trusted, and promote subordinates to form a stable psychological safety experience. At the same time, the support, resources, and tolerance given to employees by benevolent leaders will further enhance their psychological safety (Han et al., 2017). When a leader exhibits benevolent leadership and authoritarian leadership at the same time, although it can meet the different needs of subordinates, it may also lead to the cognitive dissonance of subordinates (Zhao and Guo, 2017), accelerated resource exhaustion, and weakened psychological contracts (Jiao, 2022). At this time, subordinates will not only be unable to grasp the leader's true intentions and ways of treating themselves, and thus unable to accurately position their roles (Wu and Peng, 2017), and they will also feel confused about their work due to the two different styles of instructions from the leaders. It can be seen that authoritarian-benevolent leadership is easy to make subordinates to have more uncertainty (De Cremer, 2003).

**Figure 1:**
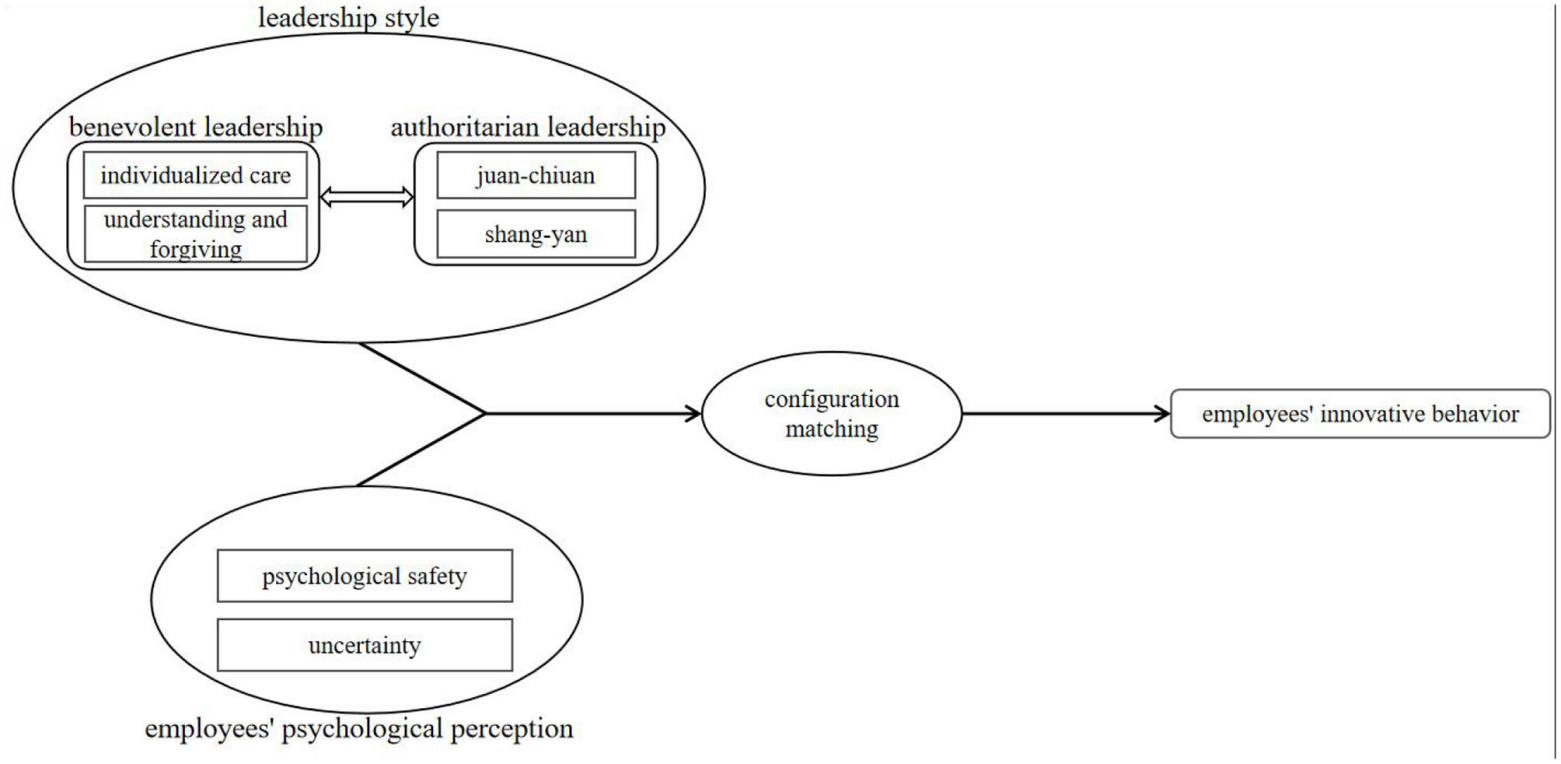
Research model of the study.

Employees' innovative behavior is the result of the combined action of multiple factors, and its formation process is a relatively complex issue. However, most of the previous studies are based on hypothesis testing or net effect analysis of the independent effect of a single influencing factor. There is a lack of research on the joint effect of multiple influencing factors. It is difficult to systematically analyze the complex formation mechanism and related paths of employees' innovative behavior based on the net effect of a single variable. The key research problem of this study is the definition of the combination of factors influencing employees' innovative behavior. Through the review of previous literature, the relationship between authoritarian-benevolent leadership and employees' psychological perceptions is not completely independent but affects each other. Authoritarian-benevolent leadership will affect employees' psychological perceptions, and employees' psychological perceptions will in turn affect authoritarian-benevolent leadership; in terms of outcome variables, the path leading to employees' innovative behavior is not unique, and there may be multiple paths. Qualitative comparative analysis is precisely a favorable tool for studying complex problems with multiple causes and effects, and it is suitable for exploring the joint effects of multiple factors that are related to the same result. From the perspective of configuration, this study combines the factor of leaders—authoritarian-benevolent leadership, and the factors of employees psychological safety and uncertainty. This study divides this leadership into four dimensions, combining the psychological safety and uncertainty of employees as influencing factors for a combined discussion to explore which combination of factors can stimulate the desired results, and what factors are the core conditions for driving the results. Therefore, the research framework of this study is constructed as shown in Figure 1.

## Materials and Methods

### Method

The qualitative comparative analysis method was proposed by the American sociologist Ragin. This method focuses on the configuration effect, and is based on technical means such as sets and Boolean algebra to analyze the formation causes, combination paths, and influence methods of complex social phenomena, and puts forward relevant practical inspirations based on the idea of set theory. QCA is suitable for small case studies under 15, medium-sized samples between 15 and 50, and large-scale samples with more than 100 cases. QCA organically integrates case-oriented qualitative methods and variable-oriented quantitative methods, considering the depth and breadth of research, and has become an important tool to effectively resolve complex causal relationships in management, marketing, and information management systems.

The reasons for choosing QCA in this study are as follows: First, QCA pays more attention to the combined influence of elements, which helps to explore the complex causal relationship between the configuration and the result formed by multiple antecedent conditions. Second, QCA has equivalence and can identify different action paths that drive employees' innovative behavior. Compared with the traditional single linear analysis method, the conclusions drawn by QCA can better explain the internal connection between complex phenomena and can better guide social practice. In this study, the fuzzy set qualitative comparative analysis method (fsQCA) in QCA is used to calibrate the analysis conditions to any numerical value between 0 and 1. The variables in this study are all continuous variables that express the degree.

### Sample and Procedures

This study uses the questionnaire survey method to investigate the employees of 14 enterprises in Beijing, Shandong, Yunnan, Zhejiang, Hubei, and other places. Specifically, through interviews, we screened out employees with direct superiors and their direct superiors who have authoritarian-benevolent leadership behaviors and took them as interviewees. After drawing on the existing mature scales, suitable survey questionnaires were distributed to them. At the same time, when collecting data, this study also noticed that it was obtained from enterprises of different natures, including state-owned enterprises, private enterprises, foreign-funded enterprises, and joint ventures. Finally, 430 valid questionnaires were recovered. The sample information is shown in Table 1.

**Table 1:** Basic characteristics of 430 interviewees.

**Variable**	**Category**	**Percent (%)**
Gender	Male	56.51%
	Female	43.49%
Age	25 years and under	4.88%
	26–35 years	64.19%
	36–45 years	25.81%
	Over 45 years	5.12%
Education	Junior college and below	16.74%
	Bachelor's degree	63.72%
	Master's degree	18.14%
	Doctoral degree	1.40%
Working age	2 years or less	9.77%
	3–5 years	21.16%
	6–10 years	28.84%
	Over 10 years	40.23%
Enterprise nature	State-owned enterprise	46.28%
	Private enterprise	33.02%
	Foreign and joint ventures	6.28%
	Others	14.42%

### Measurements

The QCA analysis divides variables into antecedent variables and outcome variables, which correspond to independent variables and dependent variables in traditional regression analysis. This study takes the two dimensions of benevolent leadership—individualized care, understanding and forgiving, and the two dimensions of authoritarian leadership—Juan-Chiuan and Shang-yan, as well as employees' psychological safety and uncertainty as antecedent variables, and employees' innovative behavior as the result variable. Data were obtained using 5-point Likert scales.

### Benevolent Leadership

Referring to the research of Cheng et al. (2000), 10 items were used for measurement, including 5 items of individualized care and 5 items of understanding and forgiving.

### Authoritarian Leadership

Referring to the research of Chou et al. (2010), 8 items were used to measure the Juan-Chiuan dimension, and 10 items were used to measure the Shang-yan dimension.

### Psychological Safety

The 5-item employee self-assessment scale developed by Liang et al. (2012) was used, with example items such as: “In my work unit, I can express my true feelings about my work.”

### Uncertainty

Using the work uncertainty questionnaire compiled by Colquitt et al. (2012), containing 4 items, example items such as: “Currently, there is a lot of uncertainty in my work.”

### Employees' Innovative Behavior

Using the scale developed by Liu et al. (2009), there are 5 items in total, such as: “In order to realize the innovative ideas of my colleagues, I often offer suggestions.”

### Reliability and Validity

This study conducts reliability and validity analysis on the 430 questionnaires collected. First, the first-hand survey data were analyzed by SPSS 26.0. The Cronbach's α value of each factor was greater than 0.8, indicating that the internal consistency was good and the reliability passed the test. Next, confirmatory factor analysis was performed using AMOS 25.0. The results showed that the χ^2^/df value was 2.575, the RMSEA value was 0.061, the CFI value was 0.905, the IFI value was 0.906, and the TLI value was 0.898. The fitting results of the model were ideal. The factor loading analysis results are shown in Table 2. The factor loading coefficients meet the requirements, the average variance extraction of each factor is >0.5, and the composite reliability is >0.7, indicating good convergent validity. Finally, the discriminant validity was tested, and the analysis results are shown in Table 3. It can be seen that the correlation coefficients between the variables are all smaller than the corresponding square root of AVE, which means that there is a certain correlation between the latent variables and a certain degree of discrimination between them. The discriminant validity of the scale data is ideal. In conclusion, the overall reliability and validity of the scale can meet the research requirements of this study (A = individualized care, B = understanding and forgiving, C = Juan-Chiuan, D = Shang-yan, E = psychological safety, F = uncertainty, G = employees' innovative behavior).

**Table 2:** Factor loading analysis results.

**Path**			**Estimate**	**AVE**	**Composite reliability**
A5	< –	A	0.815	0.714	0.926
A4	< –	A	0.847		
A3	< –	A	0.908		
A2	< –	A	0.826		
A1	< –	A	0.825		
B5	< –	B	0.902	0.7	0.921
B4	< –	B	0.851		
B3	< –	B	0.85		
B2	< –	B	0.765		
B1	< –	B	0.809		
C8	< –	C	0.67	0.569	0.913
C7	< –	C	0.772		
C6	< –	C	0.712		
C5	< –	C	0.789		
C4	< –	C	0.812		
C3	< –	C	0.775		
C2	< –	C	0.775		
C1	< –	C	0.72		
D10	< –	D	0.662	0.553	0.924
D9	< –	D	0.629		
D8	< –	D	0.516		
D7	< –	D	0.753		
D6	< –	D	0.698		
D5	< –	D	0.811		
D4	< –	D	0.738		
D3	< –	D	0.836		
D2	< –	D	0.879		
D1	< –	D	0.835		
E5	< –	E	0.903	0.661	0.907
E4	< –	E	0.778		
E3	< –	E	0.815		
E2	< –	E	0.836		
E1	< –	E	0.722		
F4	< –	F	0.767	0.581	0.846
F3	< –	F	0.865		
F2	< –	F	0.762		
F1	< –	F	0.637		
G5	< –	G	0.826	0.708	0.924
G4	< –	G	0.857		
G3	< –	G	0.884		
G2	< –	G	0.874		
G1	< –	G	0.761		

**Table 3:** Discriminant validity analysis results.

	**A**	**B**	**C**	**D**	**E**	**F**	**G**
A	0.714						
B	0.691	0.7					
C	−0.269	−0.338	0.5692				
D	0.165	0.331	0.264	0.5525			
E	0.548	0.675	−0.354	0.24	0.661		
F	−0.158	−0.152	0.578	0.269	−0.119	0.5807	
G	0.365	0.42	−0.027	0.31	0.594	0.029	0.7082
Square root of AVE	0.845	0.837	0.754	0.743	0.813	0.762	0.842

### Variable Calibration

The raw data need to be calibrated and the set membership of the research conditions and results need to be assigned before the QCA analysis is carried out. Variable calibration needs to set the following three anchor points: the threshold value that completely belongs to a certain set, the threshold value that does not belong to a certain set at all, and the intersection that exists as a watershed. After calibration, the membership of each variable set is between 0 and 1. The setting of anchor points needs to be carried out according to existing theories and the actual distribution of data of cases. Referring to the study of Jacobs and Cambré (2020), the three anchor points of the condition variables and the outcome variables were set to 5, 3.5, and 1.

## Data Result Analysis

This study used the fsQCA3.0 software to analyze the data of 430 questionnaires. In practice, the setting of frequency thresholds and consistency thresholds should comprehensively consider research objectives, analysis levels, and sample size. In this study, referring to the common standards of existing studies and combining the actual situation of this study, the frequency threshold is set according to the standard of 1.5% of the sample size, so the frequency threshold is set to 7. The consistency threshold is set to 0.80, and the PRI consistency threshold is set to 0.70 to avoid the occurrence of a “simultaneous subset relationship” and the resulting problems with the same cause but different results.

### Single Factor Necessity Analysis

Before performing configuration analysis, it is necessary to check whether a single antecedent condition is a necessary condition for the result. In this study, the threshold of consistency level, which constitutes a necessary condition for the result, is set to 0.9, and the results are shown in Table 4. It can be seen that the consistency level of the antecedent conditions of each variable is lower than 0.9, which does not constitute a necessary condition. This means that a single condition is weak in explaining the emergence of employees' innovative behavior and cannot constitute a sufficient condition. It can be seen that whether employees' innovative behavior depends on a complex configuration formed by multiple antecedent factors, and the reasons are often multidimensional, rather than determined by a single factor.

**Table 4:** Single factor necessity analysis results.

**Condition variable**		**Employees' innovative**	
		**behavior**	
		**Consistency**	**Coverage**
Leadership style	Individualized care	0.595	0.905
	~Individualized care	0.735	0.689
	Understanding and forgiving	0.835	0.822
	~Understanding and forgiving	0.541	0.764
	Juan-Chiuan	0.561	0.864
	~Juan-Chiuan	0.816	0.759
	Shang-yan	0.853	0.818
	~Shang-yan	0.554	0.813
Employees' psychological	Psychological safety	0.802	0.892
perception	~Psychological safety	0.594	0.721
	Uncertainty	0.665	0.850
	~Uncertainty	0.734	0.779

### Configuration Analysis

In this study, the fsQCA software is used to explore the configuration of employees' innovative behavior generated by the above-mentioned six antecedent conditions, and three solutions are obtained: Complex solutions (excluding logical remainders), intermediate solutions (only including logical remainders that conform to theoretical direction expectations and empirical evidence), and parsimonious solutions (including all logical remainders but no rationality assessment).

Among them, if the antecedent condition appears in the parsimonious solutions and the intermediate solutions at the same time, it is the core condition and has an important influence on the result. If the antecedent condition only appears in the intermediate solution, it is an edge condition and plays an auxiliary role. There are two key metrics for interpreting the fsQCA analysis results: Consistency, an indicator used to measure the strength of the antecedent condition is a necessary condition for the outcome variable. Coverage, the index reflects the proportion of the result samples that a configuration can explain, and is used to measure the correlation between a configuration and the outcome variable.

The relevant symbols are explained as follows: Referring to Ragin's (2009) expression, use • to indicate that the condition variable appears, and use ⊗ to indicate that the condition variable does not appear. Among them, the large circles represent the core conditions, and the small circles represent the edge conditions. A space indicates that the condition variable is irrelevant (either present or absent).

It can be seen from Table 5 that five driving paths are generated through configuration analysis, and the consistency indicators are 0.94, 0.96, 0.95, 0.97, and 0.97, indicating that the five configurations are all sufficient conditions for a high level of employees' innovative behavior. The model solution coverage is 0.72, indicating that 5 paths explain 72% of the high level of employees' innovative behavior samples. Next, this study will conduct an in-depth analysis of the five paths.

**Table 5:** Configuration analysis results.

**Condition variable**	**Configurations that produces high level of**				
	**employees' innovative behavior**				
	**➀**	**➁**	**➂**	**➃**	**➄**
Individualized care		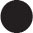	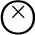	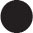	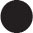
Understanding and forgiving	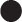	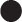		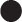	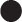
Juan-chiuan	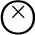		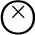	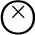	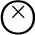
Shang-yan	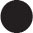	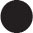	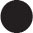	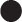	
Psychological safety	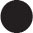	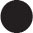	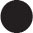		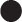
Uncertainty			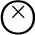	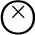	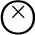
Consistency	0.936	0.959	0.949	0.966	0.966
Coverage	0.608	0.519	0.463	0.453	0.459
Unique coverage	0.033	0.049	0.017	0.018	0.024
Solution consistency	0.926				
Solution coverage	0.716				

### Understanding and Forgiving × ~ Juan-Chiuan × Shang-Yan × Psychological Safety

This configuration shows that if the leader adopts a leadership style of understanding and forgiving, non-Juan-Chiuan and Shang-yan, when the subordinates have a high level of psychological safety, employees' innovative behavior can be effectively stimulated.

First, according to the social exchange theory, benevolent leadership creates a comfortable and supportive innovation environment by endowing subordinates with favors and encourages employees to take risks, construct new thinking, try new methods, and improve their innovation performance to reward benevolent leaders. The understanding and forgiving in benevolent leadership can allow employees to try and make mistakes in terms of work tasks, provide employees with innovative development training and feedback, encourage employees to break through the boundaries of traditional practice and innovate through trial and error, and increase the probability of employees obtaining breakthrough innovations.

Second, Juan-Chiuan leaders usually do not disclose information to subordinates, and rarely authorize subordinates. They do not even allow subordinates to question their authority so all work behaviors of subordinates conform to their requirements and assumptions. Over time, subordinates will develop strong fear, lose enthusiasm for work tasks, only perform behaviors limited to the contract, and rarely produce out-of-role behaviors. Subordinates do not go beyond the scope of their responsibility to generate innovative ideas, and even if there are breakthrough ideas, they do not put them into practice.

Third, Shang-yan leadership requires high performance and maintenance of organizational norms, which will lead to high self-requirements and work engagement of subordinates, enhance their sense of identity with the leader, and then improve their subjective initiative, which is conducive to their production of more novel and effective ideas for practice. At the same time, Shang-yan leaders will also give certain authorization and guidance to employees to help employees clearly define the goals and rules of work, and reduce the feeling of losing control in the work of subordinates. This will help subordinates have confidence and ability to complete tasks, which is conducive to promoting employees' innovative behavior.

Finally, psychological safety plays a big role in individuals engaging in innovative and dedicated activities. The theory of resource conservation points out that psychological safety, as a protective resource, encourages employees to express themselves boldly, and they will not scruple or fear the negative career impact of their advice, innovation, and adventure. Therefore, when employees have a high level of psychological safety, the crisis and pressure brought by innovation risks will also be reduced, so that employees can get rid of psychological constraints and devote themselves to innovation; At the same time, psychological safety will also enable employees to break through the existing thinking and work framework, help them focus more on work innovation, generate more innovative solutions and ideas, and then increase innovation output.

### Individualized Care × Understanding and Forgiving × Shang-Yan × Psychological Safety

This configuration shows that if the leader shows a high level of individualized care and understanding and forgiving, and at the same time adopts a high level of Shang-yan leadership style, when the subordinates have a high sense of psychological safety, employees' innovative behavior can be effectively stimulated.

Individualized care in benevolent leadership can help individuals better integrate into their work roles by treating subordinates as family members, providing helping care and emotional support, and increasing organizational identity and emotional embeddedness, then the subordinates will tend to put aside the burden and take the initiative to innovate and give back to the leaders life and emotional grace.

In addition, the configuration also shows that when the leader shows high-level Shang-yan behaviors and the subordinates have a strong sense of psychological safety, the leader can consider showing individualized care and understanding and forgiving at the same time. This is because benevolent leadership is the leader's care and help in the personal and work aspects of subordinates. Combining individualized care with understanding and forgiving will promote subordinates to increase their gratitude and satisfaction, thereby promoting them to show more out-of-role behaviors. In this way, employees will be willing to take the initiative to take risks and conduct more trial and error when the work faces difficulties that need to be broken through. The Shang-yan behaviors of the leader can also effectively restrain the negative effects of high-level benevolent leadership, and reduce the possibility of employees breeding work inertia and overly obedience to the leader, which is also conducive to employee innovation.

### ~ Individualized Care × ~ Juan-Chiuan × Shang-Yan × Psychological Safety × ~ Uncertainty

This configuration shows that if the leader exhibits a low level of individualized care, at the same time exerting the Shang-yan side of authoritarian leadership and weakening the Juan-Chiuan side, when subordinates have high psychological safety and low uncertainty, employees' innovative behavior can still be effectively stimulated.

The psychological perception of uncertainty will hinder employees from making changes at work, and even affect the normal workflow. In the past, most of the theories on employees' job uncertainty believed that uncertainty was an obstacle and was not conducive to the formation of employees' willingness to innovate. This is because uncertainty means that employees are confused and doubtful about the current situation, and often have no ability and take the next step with confidence. Employees with a high level of uncertainty will feel overwhelmed when problems arise outside of the norm in the organization and feel tied down, making it difficult to have the courage to decide to change. Employees with a low level of uncertainty, on the other hand, are more determined and courageous to act and make improvements in the face of adversity and are more likely to make breakthrough innovations for individuals and organizations.

The feature of this configuration is that when employees have a high sense of psychological safety and a low sense of uncertainty, as long as the leaders maintain the Shang-yan leadership style and eliminate the Juan-Chiuan leadership style, even if they do not show individualized care and understanding and forgiving, employee innovation behavior can also be effectively stimulated. Relevant studies have shown that Shang-yan leadership and employees' psychological safety are important antecedent variables for employees' innovative behavior. Shang-yan means that leaders have high requirements for work, and this high demand is manifested as a dedicated and responsible attitude, which is very important for the organization. It is positive in this case that employees are influenced by their leaders to work harder. The high psychological safety of employees means that they think they are “safe” in the organization, their opinions and behaviors will be respected and supported by their superiors and colleagues, and they will not be easily excluded and denied, which has a great significance on whether they are inclined to take the initiative to innovate. To sum up, the combination of Shang-yan and psychological safety can become a sufficient condition to promote employees' innovative behavior.

### Individualized Care × Understanding and Forgiving × ~ Juan-Chiuan × Shang-Yan × ~ Uncertainty

This configuration shows that if the leader shows individualized care and understanding and forgiving of benevolent leadership to the subordinates, as well as exerts the Shang-yan side of authoritarian leadership and eliminates the Juan-Chiuan side, when the subordinate has a low sense of uncertainty, employees' innovative behavior can be effectively stimulated.

Leaders who combine benevolent leadership with Shang-yan leadership can not only provide subordinates with a comfortable working environment, and improve their loyalty and work input, but also improve their self-requirements and work output through high demands. When the leader displays favor and majesty in a balanced and high degree, employees are given room for correction and improvement, and at the same time, they feel that the leader has high expectations for themselves. At this time, they will positively attribute the leader's behavior. Therefore, when faced with work problems that need to be innovatively solved, out of the psychology of “return” and “self-realization,” employees will not escape the problem but will dare to face the problem and actively think about the problem to find a ”breakthrough.”

In this situation, leaders should pay attention to avoiding the Juan-Chiuan style and not taking control otherwise, it will suppress the initiative of the employees, which is not conducive to the generation of employees' innovative behavior. At the same time, the configuration also shows that as long as leaders adopt benevolent and Shang-yan leadership instead of Juan-Chiuan leadership, and pay attention to reducing employees' uncertainty, even if they do not have a high level of psychological safety, employees' innovative behavior can still be stimulated.

### Individualized Care × Understanding and Forgiving × ~ Juan-Chiuan × Psychological Safety × ~ Uncertainty

This configuration shows that when leaders adopt the benevolent leadership style and do not exhibit Juan-Chiuan behaviors, employees' innovative behavior can be effectively stimulated when subordinates have a high sense of psychological safety and a low sense of uncertainty.

Compared with the fourth driving path, it can be seen that although a Shang-yan leader can reduce uncertainty in the work of subordinates through strict requirements, clear goals, and rules, and giving instructions to subordinates, thus facilitating innovation, the creation of innovative behavior does not necessarily mean that the leader adopts a Shang-yan style. Further, benevolent leaders can promote innovation by giving enough care to their subordinates through individual attention, understanding, and forgiving, although some studies have shown that benevolent leaders have the risk of exerting a negative influence on their subordinates, which can be solved by being strict. The willingness to innovate can be stimulated even in the absence of a Shang-yan leadership style.

## Discussion

### Theoretical Implications

The theoretical significance of this study lies in the following points: (1) By dividing benevolent leadership and authoritarian leadership into four dimensions: individualized care, understanding and forgiving, Juan-Chiuan, and Shang-yan, we find out the combination of these dimensions that have a positive impact on employees' innovative behavior and identify the key conditions. There are few existing studies on China's authoritarian-benevolent leadership. The results of this research can not only enrich the existing authoritarian-benevolent leadership theories but also provide theoretical support for a better understanding of the modern transformation of the connotation of authoritarian-benevolent leadership in the context of modern enterprises to provide a certain reference for subsequent research in this field. (2) When discussing the formation of employees' innovative behavior, this study not only considers the influence of leaders but also incorporates the psychological perceptions of subordinates into the research, which verifies the status of employees' psychological perception as an important factor affecting employees' innovative behavior. This has a certain value for subsequent research on stimulating employees' active innovation. (3) This study is the first to apply the method of fuzzy set qualitative comparative analysis (fsQCA) in the study of authoritarian-benevolent leadership. Taking individualized care, understanding and forgiving benevolent leadership, Juan-Chiuan and Shang-yan of authoritarian leadership, and employees' psychological safety and uncertainty as the influencing factors, the configuration matching of the employees' psychological perceptions and authoritarian-benevolent leadership are carried out to explore its influence on employees' innovative behavior, and finally, get five configurations that can stimulate high-level employees' innovative behavior. Different from previous studies on linear causality analysis, using the fuzzy set qualitative comparative analysis method is helpful to explore the complex causal relationship between configuration and outcome formed by multiple antecedent conditions, and finding multiple different paths with the same driving effect.

### Practical Implications

Through the five configurations obtained in the above analysis, some management inspirations from organizational leaders to their subordinates can be obtained. (1) In this study, through configuration analysis, the two core conditions of individualized care, Shang-yan × psychological safety were obtained. This means that leaders can pay attention to the full display of individualized care in management practices as well as exert a Shang-yan style when employees have a high sense of psychological safety to stimulate employees' innovative behavior. (2) Among the five configurations obtained in this study, four paths combine Shang-yan with individualized care or understanding and forgiving, and there are also four paths that have the condition variable ~ Juan-Chiuan, which indicates that it is possible for leaders to adopt authoritarian-benevolent leadership when managing. But there are still a few things to note. On the one hand, this study affirms the role of benevolent leadership in inspiring employees' innovative behavior, but leaders should also pay attention to the negative effects that benevolent leadership may bring, and adopt the Shang-yan leadership to prevent subordinates from breeding inertia or overly obedience to the leader. Leaders should give full play to the positive role of Shang-yan leadership in promoting employee innovation to better meet the innovation needs of the organization and improve the innovation performance of the organization. On the other hand, leaders should also pay attention to resolutely eliminate the Juan-Chiuan side while giving full play to the Shang-yan side of authoritarian leadership, and must not adopt the Juan-Chiuan leadership style, to be “right to things and not to people.” Leaders eliminate the uncertainty of subordinates through high requirements and clear instructions, and on this basis, combine individualized care and understanding and forgiving according to the situation to achieve the best management effect. (3) This study also shows that two psychological variables of employees—psychological safety and uncertainty are important factors that determine their behavior. Specifically, in the five configurations, each occurrence of psychological safety is high, and each occurrence of uncertainty is low, which indicates that high psychological safety and low uncertainty are important conditions for employees' innovative behavior. Leaders should pay attention to enhancing the psychological safety of subordinates and weakening their sense of uncertainty in management.

### Limitations and Future Directions

There are still some shortcomings in this study. First of all, this study mainly focuses on the influence of authoritarian-benevolent leadership and employees' psychological perception of employees' innovative behavior and analyzes the configuration formed by the antecedent conditions of the outcome variable of high-level employees' innovative behavior. It does not analyze the low-level employees' innovative behavior. The research on employees' innovation behavior is still not perfect. Second, this study selects psychological safety and uncertainty as condition variables at the level of employees' psychological perception. Although these two variables have received extensive attention in previous studies, indicating that they are highly correlated with employees' innovative behavior, these are not the only two psychological variables of employees. There are other psychological variables that deserve attention and discussion in the process of interaction between subordinates and superiors in the organization.

In the future, we can continue to use the QCA method to conduct in-depth analysis and research on the influencing factors of employees' innovation behavior, especially to obtain the antecedent condition configuration of low-level employees' innovative behavior, and compare it with the results of this study to enrich and improve theories about employee innovation and gain enlightenment for management practice. In addition, regarding employees' psychological perception, we can also broaden our thinking, discover other important psychological variables, and explore the role of existing important psychological variables on employees' innovative behavior, which requires further research by researchers to explore other mechanisms.

## Conclusion

The main conclusions of this study are as follows: the combination of individualized care, understanding, and forgiving of benevolent leadership, and Shang-yan of authoritarian leadership can effectively stimulate employees' innovative behavior; Juan-Chiuan leadership is not conducive to the production of employees' innovative behavior; employees' high psychological safety and low uncertainty are important conditions for promoting employee innovation.

## Data Availability Statement

The raw data supporting the conclusions of this article will be made available by the authors, without undue reservation.

## Ethics Statement

Ethical review and approval was not required for the study on human participants in accordance with the local legislation and institutional requirements. Written informed consent from the patients/ participants or patients/participants legal guardian/next of kin was not required to participate in this study in accordance with the national legislation and the institutional requirements.

## Author Contributions

LM mainly carried out theoretical analysis and model construction for the study. TL was responsible for theoretical analysis and literature search. MY carried out model construction for the study. SW was responsible for submitting comments for later revisions of the manuscript. All authors contributed to the structure and design of the study and completed the revision phase together.

## Conflict of Interest

The authors declare that the research was conducted in the absence of any commercial or financial relationships that could be construed as a potential conflict of interest.

## Publisher's Note

All claims expressed in this article are solely those of the authors and do not necessarily represent those of their affiliated organizations, or those of the publisher, the editors and the reviewers. Any product that may be evaluated in this article, or claim that may be made by its manufacturer, is not guaranteed or endorsed by the publisher.
